# Association Between Serum per- and Polyfluoroalkyl Substances and Iron Status Biomarkers in a Representative Sample of U.S. Adults: NHANES 2013–2018

**DOI:** 10.3390/life15081274

**Published:** 2025-08-12

**Authors:** Wei-Jie Wang, Yu-Ling Lin, Ta-Chen Su, Chikang Wang, Chien-Yu Lin

**Affiliations:** 1 Division of Nephrology, Department of Internal Medicine, Lo-Sheng Sanatorium and Hospital, Ministry of Health and Welfare, Taoyuan 330, Taiwan; mrwwj.tw@lslp.mohw.gov.tw; 2 Department of Biomedical Engineering, Chung Yuan Christian University, Taoyuan 300, Taiwan; 3 Department of Nephrology, Hsinchu Cathay General Hospital, Hsinchu 300, Taiwan; cgh07022@cgh.org.tw; 4 Department of Healthcare Management, Yuanpei University of Medical Technology, Hsinchu 300, Taiwan; 5 Institute of Environmental and Occupational Health Sciences, College of Public Health, National Taiwan University, Taipei 100, Taiwan; tachensu@gmail.com; 6 Department of Internal Medicine, National Taiwan University Hospital, Taipei 100, Taiwan; 7 Department of Internal Medicine, College of Medicine, National Taiwan University, Taipei 100, Taiwan; 8 Department of Internal Medicine, Tungs’ Taichung MetroHarbor Hospital, Taichung 435, Taiwan; 9 Department of Environmental Engineering and Health, Yuanpei University of Medical Technology, Hsinchu 300, Taiwan; ckwang@mail.ypu.edu.tw; 10 Department of Internal Medicine, En Chu Kong Hospital, No. 399, Fuxing Road, Sanxia District., New Taipei City 237, Taiwan; 11 School of Medicine, Fu Jen Catholic University, New Taipei City 242, Taiwan

**Keywords:** ferritin, iron homeostasis, national health and nutrition examination survey (NHANES), per- and polyfluoroalkyl substances (PFAS), serum iron, transferrin receptor

## Abstract

**Purpose:** Per- and polyfluoroalkyl substances (PFAS) comprise a class of man-made compounds widely utilized in manufacturing everyday consumer products. Experimental studies indicate that PFAS may interfere with iron regulation by hindering absorption or inducing oxidative stress. Nonetheless, epidemiological studies examining the association between PFAS exposure and a broad spectrum of iron-related biomarkers remain scarce. **Approach and Results:** In this study, data from the 2013–2018 National Health and Nutrition Examination Survey (NHANES) were analyzed, which included 5050 adults aged 18 and older. The relationships between six PFAS compounds, oral iron intake, and a comprehensive set of markers of iron homeostasis, including serum iron, unsaturated iron-binding capacity (UIBC), total iron-binding capacity (TIBC), transferrin saturation, ferritin, and transferrin receptor levels, were examined. Our findings revealed a negative association between both individual and total PFAS (sum of six PFAS) levels and oral iron intake. Additionally, serum iron and transferrin saturation levels exhibited significant positive correlations with all PFAS compounds, whereas ferritin was positively correlated with all PFAS compounds except *n*-perfluorooctanoic acid (*n*-PFOA). UIBC and transferrin receptor showed significant negative correlations with all PFAS compounds, while TIBC was significantly negatively correlated with *n*-perfluorooctane sulfonic acid (*n*-PFOS), perfluoromethylheptane sulfonic acid isomers (*sm*-PFOS), perfluorohexane sulfonic acid (PFHxS), and the total PFAS. **Conclusions:** Higher PFAS exposure was associated with altered iron status biomarkers While this cross-sectional study cannot establish causality, the observed associations raise the possibility that PFAS exposure may influence iron absorption. These findings emphasize the need for additional research into the potential impact of PFAS exposure on iron homeostasis.

## 1. Introduction

Per- and polyfluoroalkyl substances (PFAS), a class of human-made chemicals characterized by strong carbon–fluorine bonds, have been incorporated into a wide array of everyday products since the mid-20th century [1]. Due to their extensive use and persistence in the environment, PFAS have become widespread across various habitats and species worldwide [2]. Human exposure occurs through multiple pathways, including ingestion of contaminated food and water, contact with food packaging materials, inhalation of indoor air, and household dust. Of these, dietary intake—especially the consumption of fish—represents the predominant route of PFAS exposure [3].

Exposure to PFAS in humans has been associated with a wide range of adverse health outcomes, such as carcinogenesis, hepatic toxicity, thyroid dysfunction, immune system impairment, and developmental delays in children [4]. Globally, a variety of regulatory measures have been adopted, including production and usage prohibitions within the European Union and the establishment of exposure thresholds and waste management protocols in the United States [5]. PFAS levels in humans have declined since 2000 due to regulatory actions and voluntary phase-outs of certain PFAS compounds by manufacturers [6,7,8].

Iron is vital for health, as it helps transport oxygen in the blood, supports energy production, assists in DNA synthesis, and aids in immune function. Deficiency can cause anemia and fatigue, while excess iron can lead to toxicity [9]. In evaluating iron status, several key indicators offer a comprehensive assessment of iron levels in the body. Serum iron represents the fraction of iron currently bound to transferrin and available for metabolic use. Unsaturated iron-binding capacity (UIBC) quantifies transferrin’s unoccupied binding sites; total iron-binding capacity (TIBC) equals serum iron + UIBC and captures transferrin’s full transport potential, while transferrin saturation expresses the percentage of filled sites [10]. Ferritin, an intracellular storage protein, reflects systemic iron reserves [11]. The transferrin receptor, an iron-import membrane glycoprotein, indexes cellular iron demand by mediating uptake of transferrin–iron complexes [12].

PFAS may potentially disrupt iron homeostasis in humans through various mechanisms. Exposure to PFAS can disrupt bile acid profiles [13,14], potentially altering intestinal pH and impacting iron absorption. Additionally, PFAS may damage intestinal tissue, leading to increased permeability and changes in nutrient absorption [15]. Furthermore, PFAS could influence the gut microbiota, which plays a role in iron metabolism, thereby affecting iron absorption [16,17]. Epidemiological studies have revealed a link between PFAS exposure and biomarkers of iron status. Analysis of National Health and Nutrition Examination Survey (NHANES) data from 2005–2012 and 2007–2018 revealed a positive association between PFAS exposure and serum iron levels, though no significant relationship with ferritin levels was observed. Oxidative stress is thought to play a role in this association [18,19]. However, these studies have primarily focused on the relationship between PFAS exposure and serum iron and ferritin, rather than a comprehensive assessment of iron status biomarkers. Our recent longitudinal study in Taiwan following 502 participants found positive associations between PFAS exposure and levels of serum iron, ferritin, and transferrin saturation. However, the study had a relatively small sample size and did not account for dietary iron intake, which is a key factor in evaluating iron homeostasis [20].

To address the knowledge gap, we analyzed NHANES data from 2013 to 2018. This dataset encompasses serum levels of six PFAS compounds, oral iron intake, and a comprehensive set of iron homeostasis biomarkers, including serum iron, UIBC, TIBC, transferrin saturation, ferritin, and transferrin receptor. The aim of this study was to investigate the relationship between PFAS exposure and biomarkers of iron homeostasis in the U.S. adult population.

## 2. Materials and Methods

### 2.1. Study Population

**Figure 1:**
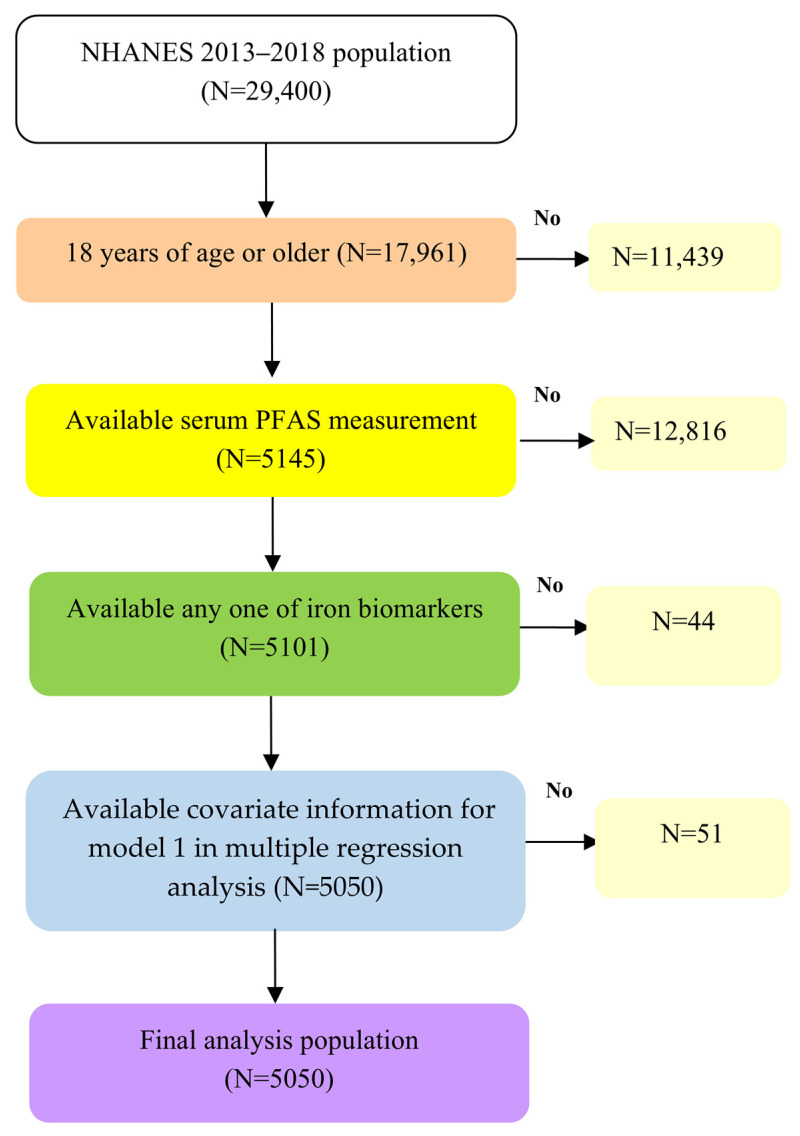
Flow chart algorithm.

NHANES is a comprehensive biennial program that evaluates the health and nutritional status of the U.S. population through interviews, physical examinations, and laboratory tests. Comprehensive details regarding survey procedures and consent documentation are accessible on the NHANES website [21]. This study utilized data from the NHANES 2013–2018 cycles, focusing on individuals aged 18 and older with available PFAS measurements, at least one iron status biomarker, and relevant demographic information, including age, sex, ethnicity, and BMI. The final analysis included 5050 participants, with the selection process visually represented in Figure 1.

### 2.2. Measurement of Serum PFAS Levels

From 2013 to 2018, NHANES analyzed serum levels of nine PFASs in one-third of the study population across three survey cycles. In this study, six types of PFASs detected in more than 60% of participants were included: *n*-perfluorooctanoic acid (*n*-PFOA), *n*-perfluorooctane sulfonic acid (*n*-PFOS), perfluoromethylheptane sulfonic acid isomers (*sm*-PFOS), perfluorononanoic acid (PFNA), perfluorohexane sulfonic acid (PFHxS), and perfluorodecanoic acid (PFDeA). The detection rates for these compounds were 99.3%, 99.3%, 98.7%, 96.6%, 98.7%, and 79.8%, respectively. Serum samples were analyzed at the CDC using online solid-phase extraction combined with high-performance liquid chromatography (HPLC) and tandem mass spectrometry (Chromolith® HighResolution RP-18e columns, Merck KGaA, Darmstadt, Germany). A 50 μL aliquot of serum was acidified and processed using a column-switching system that retained target analytes on a solid-phase extraction column. Compounds were separated from matrix components by HPLC and quantified using TurboIonSpray ionization in negative-ion mode. Detailed laboratory methods are available in the PFAS Laboratory Procedure Manual of NHANES [22]. PFAS have a laboratory limit of detection (LOD) of 0.10 ng/mL. Values below the LOD were replaced with the LOD divided by the square root of two.

### 2.3. Measurement of Biomarkers of Iron Status

In the NHANES 2013–2018 dataset, serum iron levels were assessed using the Roche Cobas 6000 analyzer (Roche Diagnostics, Indianapolis, IN, USA) with a three-step FerroZine reagent method [23]. For the NHANES 2017–2018 cycle, UIBC was measured with the same analyzer. TIBC was derived by adding serum iron to UIBC, while transferrin saturation was determined by dividing serum iron by TIBC and multiplying by 100 [24]. In the NHANES 2015–2018 dataset, serum ferritin was measured using a sandwich assay on the Roche Cobas^®^ e601 analyzer (Roche Diagnostics, Indianapolis, IN, USA) [25]. Additionally, transferrin receptor levels were determined using a particle-enhanced immunoturbidimetric assay on the Roche Cobas^®^ c501 clinical analyzer (Roche Diagnostics, Indianapolis, IN, USA) during the same period [26]. The reported precision reflects the resolution of the respective assays: ferritin and transferrin receptor have LODs of 0.5 ng/mL and 0.5 mg/L, respectively, while the LODs for serum iron and UIBC are 5 µg/dL and 17 µg/dL. Values below the detection limit were replaced with the LOD divided by √2. This study included participants aged 18 years and older.

### 2.4. Covariates

Sociodemographic characteristics—including age, gender, and racial/ethnic background—were retrieved from the NHANES dataset. Smoking behavior was classified into three categories: current smokers, individuals exposed to secondhand smoke, and non-smokers, using self-reported data from the tobacco use questionnaire [27]. Alcohol consumption was evaluated by asking participants whether they had consumed at least 12 alcoholic drinks over the past 12 months, with responses dichotomized accordingly. Mean dietary iron intake was derived from participants’ reported consumption across two 24 h dietary recall interviews. Intake of iron-containing supplements was estimated by averaging usage data from two consecutive days and incorporating information obtained from a 30-day supplement use questionnaire. Total oral iron intake was computed by summing iron intake from both dietary sources and supplementation [28]. Anemia management status was identified through participants’ responses to a survey question regarding any treatment received for anemia within the preceding three months. Definitions of body mass index (BMI), diabetes, and chronic kidney disease in this analysis were informed by widely accepted criteria established in prior scientific literature [29,30].

### 2.5. Statistics

Comparisons of PFAS levels across the three NHANES cycles were conducted using the Kruskal–Wallis H test. Sampling weights were applied following NHANES protocols [31]. To investigate the relationship between PFAS exposure and iron status, linear regression with complex sampling was performed. Two models were utilized for covariate adjustment: Model 1 adjusted for age, gender, ethnicity, and BMI, while Model 2 further included adjustments for oral iron intake, chronic kidney disease, diabetes, and anemia treatment. Statistical significance was defined by requiring results to be significant in both models. To gain a more comprehensive understanding of potential health risks, the combined concentration of six types of PFAS was calculated to evaluate their overall correlation with iron status [13]. Given the non-Gaussian distribution, the natural logarithm (ln) transformation was applied to the PFAS data for analysis. To evaluate the dose–response relationship, relationships between iron status biomarkers across the sum of PFAS quartiles were analyzed. To explore the potential for non-linear dose–response relationships, we conducted additional regression analyses incorporating both the sum of PFAS exposure term and its squared term. Further analysis was also performed within distinct subpopulations, incorporating age, sex, ethnicity, and binary categories of continuous variables (BMI, oral iron intake) to explore the relationships between PFAS and iron status biomarkers. Statistical analyses were performed using IBM SPSS Statistics version 30 (IBM Corp., Armonk, NY, USA), with the significance threshold set at *p* < 0.05 to assess statistical significance.

## 3. Results

The participants in the study had an average age of 49 ± 19 years, with ages ranging from 18 to 80. The mean daily oral iron intake was 17 ± 15 mg. Importantly, 69.3% of the participants had iron intake levels below the Recommended Dietary Allowance (RDA) [32]. The baseline characteristics of study participants, along with the mean (SD), geometric mean, and ranges of serum PFAS, daily iron intake, and iron status biomarkers, are presented in Table 1 and Table 2. Supplemental Table S1 shows the correlation coefficients among different ln-PFAS concentrations. The Spearman correlation matrix indicates strong positive associations between various PFAS compounds. Notably, *n*-PFOS and *sm*-PFOS have the highest correlation, with a Spearman’s coefficient of 0.821 (*p* < 0.001). Supplemental Table S2 displays the analysis of PFAS levels across three NHANES cycles. The findings reveal substantial reductions in all six PFAS levels from 2013 to 2018, with *p*-values below 0.001 for each. This indicates a general trend of declining PFAS concentrations in the studied population over the observed period.

Table 3 presents the associations between various PFAS concentrations and iron-related biomarkers. Oral iron intake exhibited significant negative correlations with *n*-PFOA, *n*-PFOS, *sm*-PFOS, PFNA, PFHxS, PFDeA, and the sum of PFAS, with Adj. β values ranging from −1.05 to −1.77 and all *p*-values below 0.006. Serum iron levels showed significant positive correlations with all PFAS compounds, with Adj. β values between 3.50 and 5.79 and *p*-values under 0.004 in Model 1. These associations remained significant but slightly attenuated in Model 2. UIBC demonstrated significant negative correlations with all PFAS compounds in both models, with Adj. β values ranging from −10.94 to −17.83 and all *p*-values below 0.004 in Model 2. TIBC was significantly negatively correlated with *n*-PFOS, *sm*-PFOS, PFHxS, and the sum of PFAS, with Adj. β values up to −9.70 and *p*-values under 0.005 in Model 2. Transferrin saturation showed significant positive correlations with all PFAS compounds, with Adj. β values up to 2.90 and all *p*-values below 0.006 in Model 2. Ferritin was positively correlated with all PFAS compounds except *n*-PFOA, with Adj. β values up to 20.19 and *p*-values below 0.021 in Model 2. Finally, transferrin receptor levels showed significant negative correlations with all PFAS compounds, with Adj. β values up to −0.57 and all *p*-values below 0.008 in Model 2.

**Figure 2:**
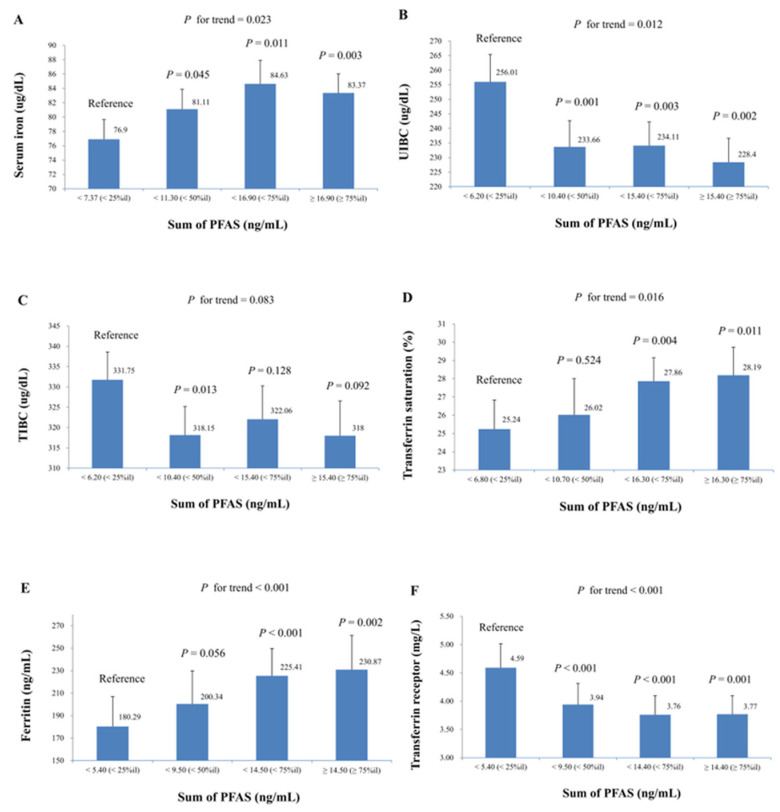
Mean (SE) of iron biomarkers across quartiles of sum of PFAS in multiple linear regression models (adjusted for model 2), with results weighted for sampling strategy. (**A**) Serum iron, (**B**) UIBC, (**C**) TIBC, (**D**) transferrin saturation, (**E**) ferritin, and (**F**) transferrin receptor.

Figure 2 summarizes the mean (SE) of iron biomarkers across quartiles of the sum of PFAS concentrations, analyzed using multiple linear regression models adjusted according to Model 2. The study found a significant increase in serum iron, transferrin saturation, and ferritin levels with increasing quartiles of the sum of PFAS. The trend *p*-values were 0.023 for serum iron, 0.016 for transferrin saturation, and <0.001 for ferritin. Additionally, a significant decrease in UIBC and transferrin receptor levels was observed across increasing quartiles of the sum of PFAS, with trend *p*-values of 0.012 and <0.001, respectively. To further explore the dose–response patterns, potential non-linear associations between PFAS exposure and iron status biomarkers were also evaluated. For serum iron, UIBC, TIBC, transferrin saturation, and ferritin, only the linear term of PFAS exposure remained statistically significant, suggesting that a linear model provided the best fit for these biomarkers. In contrast, the relationship between transferrin receptor and the ln-sum of PFAS levels exhibited a significant non-linear pattern. Both the linear (β = −1.37; SE = 0.30; *p* < 0.001) and squared (β = 0.21; SE = 0.07; *p* = 0.003) terms were significant, indicating a U-shaped association. As illustrated in Figure S1, the scatter plot of predicted values reveals this U-shaped pattern, suggesting that individuals with both low and high transferrin receptor levels tend to have higher cumulative PFAS exposure.

Table 4 displays the regression coefficients (SE) for the association between iron status biomarkers and the ln-transformed sum of PFAS for each unit increase within the defined subpopulations. For gender, men showed decreases in UIBC and transferrin receptor levels and an increase in transferrin saturation, while women had significant changes across all biomarkers. Age-specific results indicated the strongest associations in the 18–40 age group, significant for serum iron, UIBC, TIBC, and transferrin receptor. Ethnicity analysis showed significant associations across almost all biomarkers for non-Hispanic whites and other ethnic groups, except for TIBC in non-Hispanic whites and ferritin in other ethnic groups. BMI stratification revealed significant associations across almost all biomarkers, with the exceptions being TIBC for BMI < 25 and ferritin for BMI ≥ 25. Total iron intake analysis revealed significant associations across almost all biomarkers for both lower and higher iron intake, except for TIBC and ferritin in the lower intake group and transferrin saturation in the higher intake group.

## 4. Discussion

In our current study, elevated PFAS levels were associated with increased serum iron, transferrin saturation, and ferritin, while UIBC, TIBC, and transferrin receptor levels were lower. This is the first study to explore the relationship between serum PFAS levels and a comprehensive set of iron homeostasis biomarkers in a nationally representative sample of U.S. adults. If these associations imply causation, it suggests that PFAS exposure could enhance iron availability in the body, potentially influencing iron metabolism and balance.

Our results indicate a general trend of decreasing PFAS levels in the studied population over the observed period, which aligns with previous reports from the NHANES from 1999 to 2018 [8]. Similarly, a decade-long surveillance of Korean adults (2006–2015) recorded steadily rising PFAS burdens up to 2013, followed by a subsequent decline [6]. Additionally, a comprehensive study of blood samples from the Danish population, spanning 1988 to 2021, revealed that PFOS and PFOA levels increased from 1988 until the late 1990s, after which they steadily declined through 2021 [7]. The significant decrease in PFAS levels is likely a result of regulations enacted since the early 2000s [33]. In the current study, 69.3% of participants had iron intake below the RDA, consistent with prior studies indicating that a substantial portion of the U.S. population fails to meet iron intake guidelines. This trend is likely driven by changes in dietary patterns, including the increasing adoption of vegetarian lifestyles, decreased red meat intake, and the growing consumption of processed foods [34]. To mitigate this concern, it is essential to promote awareness of iron-rich dietary sources, especially among individuals adhering to restrictive eating patterns, to support sufficient iron consumption.

Our analysis also revealed that higher concentrations of serum PFAS were linked to reduced oral iron consumption, as estimated through self-reported dietary survey data. Human exposure to PFAS primarily occurs through oral intake, with common sources including contaminated water, fish and shellfish, food packaged in materials treated with PFAS (such as fast food wrappers and microwave popcorn bags), and some processed foods [35]. These sources differ from foods that are typically high in iron, such as red meat, poultry, beans, dark leafy greens, and fortified cereals [36]. In addition to differences in food sources, socioeconomic factors, lifestyle choices, and regional dietary variations could also impact both PFAS exposure and iron intake.

Numerous epidemiological investigations have explored the link between PFAS exposure and iron status biomarkers. Previous NHANES studies have suggested that the positive correlation between PFAS and serum iron was due to inflammation and oxidative stress [18,19]. A cross-sectional analysis of NHANES data from 2005 to 2012, encompassing 6652 adults, found a notable positive correlation between serum PFAS levels, serum iron, and markers of inflammation and oxidative stress. However, no association was detected with serum ferritin [18]. Another study using NHANES data from 2007 to 2018, with 7773 and 5933 participants in different models, revealed a positive correlation between serum PFAS levels and serum iron. Meanwhile, the dietary inflammatory index influenced this relationship [19]. Additionally, our longitudinal study conducted in Taiwan followed 502 adolescents and young adults aged 12 to 30 to evaluate the association between PFAS exposure and serum iron, ferritin, TIBC, and transferrin saturation. The study found positive associations between several PFAS compounds and levels of ferritin, serum iron, and transferrin saturation [20]. However, none of these studies explored the relationship between PFAS exposure and a comprehensive set of iron biomarkers, nor did they account for dietary iron intake. In the current study, a comprehensive set of iron biomarkers was evaluated while controlling for oral iron intake. The results showed that higher PFAS levels were associated with increased serum iron, transferrin saturation, and ferritin, along with decreased UIBC, TIBC, and transferrin receptor levels. Since ferritin levels rise while transferrin saturation decreases in response to inflammation, our results challenge the explanation based solely on increased inflammation and oxidative stress [37]. Our findings align more closely with the results of our long-term study in Taiwan [20]. Based on our findings, increased iron absorption due to PFAS may be a contributing mechanism. This hypothesis is further supported by a study of 821 pregnant women in China, which discovered a positive correlation between PFAS levels in cord blood and hematocrit (Hct)/hemoglobin levels, with iron supplementation intensifying this relationship [38]. Additionally, our longitudinal study in Taiwan identified notable interactions between serum transferrin saturation levels and the relationship between PFNA and Hct [20]. These findings suggest that PFAS may have a synergistic effect with iron intake on red blood cell production, providing stronger evidence that PFAS could enhance iron absorption.

Our research suggests that PFAS might increase iron absorption; however, the interaction between PFAS compounds and the biological pathways governing iron transport and homeostasis may involve several potential mechanisms. Iron levels are primarily regulated at the absorption stage, which occurs mainly in the small intestine by intestinal enterocytes [39]. Iron absorption from non-heme sources can be influenced by several factors, including duodenal pH, plant-derived absorption inhibitors like phytates and polyphenols, and the presence of enhancing nutrients such as ascorbic acid [39]. Both laboratory and population-based research have shown that PFAS exposure alters bile acid composition and metabolism [13,14]. These changes in bile acid profiles can indirectly affect iron absorption by altering the pH in the small intestine. An acidic pH helps keep iron in its soluble form, making ascorbic acid more effective at enhancing absorption, potentially improving iron absorption. However, it remains unclear whether PFAS-induced changes in bile acid profiles lead to a more acidic intestinal environment. Additionally, PFAS can impair the tight junctions in intestinal tissues and induce pathological changes in intestinal epithelial cells. As a result, these disruptions can lead to increased intestinal permeability and altered ion transport. Increased gut permeability may alter the absorption of nutrients, including iron [15].

In addition, the gut microbiome may influence the relationship between PFAS and iron absorption. Microorganisms in the gut require iron to carry out essential biological functions, including DNA replication, energy metabolism, and enzymatic activity [40]. Certain gut microbes may suppress iron absorption in the intestines by modulating hypoxia-inducible factor signaling pathways [16]. On the contrary, one study found that certain bacterial strains, such as Lactobacillus plantarum, can increase iron absorption [17]. Epidemiological studies have found that PFAS exposure can alter the composition of gut microbiota and destabilize the gut environment. These changes may play a role in PFAS-related insulin resistance, obesity, and neurodevelopmental damage [41,42,43]. Another study analyzing 25 Lactobacillus strains found that Lactobacillus plantarum CCFM738 exhibited the strongest binding affinity to PFOA [44]. Furthermore, the change of gut microbiome can also affect gut permeability, which could also increase nutrient absorption, including iron [45]. In summary, changes in gut microbiota caused by PFAS may directly or indirectly affect iron absorption. Although no studies have yet proven that PFAS-induced changes in gut microbiota increase intestinal iron absorption, this is a potential mechanism that requires further research to confirm.

Our study revealed a more pronounced negative correlation between PFAS exposure and iron status biomarkers in women. Previous studies have indicated that PFAS can interfere with normal reproductive function in women by affecting hormone secretion [46]. Additionally, animal studies indicate that PFAS exposure leads to sex-specific effects, such as increased bile acid levels [47]. Women also regularly lose iron through menstruation, which influences their iron regulation in ways that differ from men. The noted differences between sexes could stem from a mix of hormonal and metabolic factors. Our study also identified age-specific results, with the strongest associations observed in the 18–40 age group. Younger individuals generally have fewer confounding factors affecting iron status, such as chronic diseases and inflammation, compared to older individuals, which may confound the impact of PFAS on iron status [48]. Stronger associations between PFAS exposure and several iron status biomarkers—including serum iron, UIBC, transferrin saturation, and transferrin receptor—were also noted among participants with lower total iron intake. These findings may be explained by compensatory physiological mechanisms in low-iron conditions, where decreased hepcidin levels enhance ferroportin-mediated iron absorption [49]. PFAS exposure may interfere with these adaptive pathways and lead to a more pronounced effect in iron metabolism among individuals with lower baseline iron intake.

Recognizing both the strengths and limitations of this study is crucial. A major strength is that it is the first to examine the connection between serum PFAS levels and a broad array of iron homeostasis biomarkers. The analysis also accounted for numerous variables from the NHANES database, including oral iron intake, which plays a significant role in iron status biomarkers. Furthermore, our analysis offers valuable insights applicable to the wider American adult population. However, the study has certain limitations. The cross-sectional design of the NHANES database presents intrinsic limitations. Another important limitation is that dietary iron intake was assessed using self-reported 24 h dietary recalls, which are subject to recall bias and may not accurately reflect long-term iron intake. Additionally, the analysis did not account for potential co-exposure to other environmental toxicants that may also influence iron metabolism and correlate with PFAS exposure. This unmeasured confounding may have affected the observed associations. Future studies should incorporate multi-pollutant models to better disentangle the independent effects of PFAS from those of other co-occurring environmental exposures.

## 5. Conclusions

Our study employed a nationally representative sample from the U.S. and is the first to examine the relationship between serum PFAS levels and a comprehensive array of iron status biomarkers. Although the findings suggest a potential association between PFAS exposure and altered iron homeostasis, the cross-sectional design of this study precludes causal inference. Therefore, the observed associations should be interpreted with caution. Additional research is necessary to unravel the intricate interactions between PFAS exposure and iron homeostasis. Such investigations could guide health policies on PFAS regulation and contribute to protecting public health.

## Figures and Tables

**Table 1:** Demographic data in the studied population (N = 5050).

Variables	N	%
Age (year)		
18–39	1798	35.6
40–59	1584	31.4
≥60	1668	33.0
Men	2416	47.8
Ethnicity		
Mexican-American	771	15.3
Other Hispanic	536	10.6
Non-Hispanic white	1852	36.7
Non-Hispanic black	1080	21.4
Non-Hispanic Asian	599	11.9
Other ethnicity	212	4.2
Smoking status		
Active smokers	1163	23.0
Environmental tobacco smoke	802	15.9
Non-smokers	3085	61.1
≥12 alcoholic drinks/year	2733	54.1
Diabetes Mellitus	970	19.2
Chronic renal failure	269	5.3
Body mass index	3598	71.2
<25	1452	28.8
25–29	1594	31.2
≥30	2004	40.0

**Table 2:** Summary statistics of PFAS concentrations, oral iron intake, and iron status biomarkers in the studied population.

Variables	N	Mean ± SD	Geometric Mean	Minimum	Maximum
PFAS (ng/mL)					
*n*-PFOA	5050	1.98 ± 2.36	1.49	0.07	85.20
*n*-PFOS	5050	5.74 ± 19.22	3.51	0.07	1270.00
*sm*-PFOS	5050	2.05 ± 2.62	1.38	0.07	133.00
PFNA	5050	0.77 ± 0.73	0.57	0.07	16.30
PFHxS	5050	1.80 ± 2.18	1.19	0.07	48.80
PFDeA	5050	0.30 ± 0.86	0.19	0.07	51.30
Oral iron intake (mg/day)	4031	17.18 ± 14.78	14.18	0.91	369.42
Iron status biomarkers					
Serum iron (ug/dL)	5044	84 ± 36	76	7	325
UIBC (ug/dL)	1670	236 ± 65	227	12	540
TIBC (ug/dL)	1669	324 ± 53	320	169	605
Transferrin saturation (%)	1669	27.63 ± 11.96	25	3	92
Ferritin (ng/mL)	2147	131.7 ± 160.6	77.1	1.8	2030.0
Transferrin receptor (mg/L)	2139	3.5 ± 2.2	3.2	1.2	39.5

**Table 3:** Linear regression coefficients (standard error) of biomarkers of iron status with a unit increase in ln-PFAS in multiple linear regression models, with results weighted for sampling strategy.

	PFAS (ng/mL)		*n*-PFOA	*n*-PFOS	*sm*-PFOS	PFNA	PFHxS	PFDeA	Sum of PFAS
		N	Adj. β (SE)	Adj. β (SE)	Adj. β (SE)	Adj. β (SE)	Adj. β (SE)	Adj. β (SE)	Adj. β (SE)
Oral iron intake (mg/day)	Model 1	4031	−1.07 (0.33)	−1.54 (0.41)	−1.67 (0.42)	−1.72 (0.45)	−1.05 (0.36)	−1.33 (0.44)	−1.77 (0.46)
	*p*		0.002	0.001	<0.001	<0.001	0.006	0.004	<0.001
Serum iron (ug/dL)	Model 1	5044	5.66 (0.92)	3.94 (0.96)	4.69 (1.12)	3.50 (1.15)	4.05 (0.92)	4.28 (0.91)	5.79 (1.11)
	*p*		<0.001	<0.001	<0.001	0.004	<0.001	<0.001	<0.001
	Model 2	4026	4.36 (0.99)	3.39 (1.03)	3.80 (1.16)	2.88 (1.13)	3.02 (0.98)	3.90 (1.10)	4.91 (1.18)
	*p*		<0.001	0.002	0.002	0.014	0.004	0.001	<0.001
UIBC (ug/dL)	Model 1	1670	−12.11 (2.42)	−13.47 (2.39)	−14.57 (2.66)	−9.58 (3.05)	−11.98 (2.67)	−12.78 (2.30)	−16.36 (2.69)
	*p*		<0.001	<0.001	<0.001	0.007	<0.001	<0.001	<0.001
	Model 2	1332	−12.47 (2.55)	−14.58 (3.37)	−15.23 (2.99)	−10.94 (3.26)	−11.45 (2.74)	−13.45 (2.94)	−17.83 (3.36)
	*p*		<0.001	0.001	<0.001	0.004	0.001	<0.001	<0.001
TIBC (ug/dL)	Model 1	1669	−4.00 (2.66)	−7.92 (1.89)	−8.94 (1.69)	−5.72 (2.92)	−7.22 (1.85)	−6.32 (2.80)	−8.96 (2.28)
	*p*		0.154	0.001	<0.001	0.069	0.001	0.039	0.001
	Model 2	1331	−4.23 (2.74)	−8.25 (2.48)	−9.70 (1.94)	−6.08 (3.13)	−6.85 (2.00)	−5.95 (3.40)	−9.66 (2.52)
	*p*		0.143	0.005	<0.001	0.071	0.004	0.101	0.002
Transferrin saturation (%)	Model 1	1669	2.55 (0.33)	2.13 (0.46)	2.19 (0.63)	1.44 (0.42)	1.84 (0.47)	2.34 (0.30)	2.70 (0.52)
	*p*		<0.001	<0.001	0.003	0.004	0.001	<0.001	<0.001
	Model 2	1331	2.57 (0.39)	2.31 (0.57)	2.20 (0.69)	1.75 (0.43)	1.75 (0.52)	2.54 (0.38)	2.90 (0.64)
	*p*		<0.001	0.001	0.006	0.001	0.004	<0.001	<0.001
Ferritin (ng/mL)	Model 1	2147	11.15 (6.05)	14.51 (5.10)	16.70 (4.49)	15.80 (3.98)	12.61 (5.26)	16.45 (3.55)	18.74 (6.22)
	*p*		0.075	0.008	0.001	<0.001	0.023	<0.001	0.005
	Model 2	1695	12.62 (7.13)	15.40 (6.33)	17.64 (4.93)	16.97 (5.53)	13.27 (5.21)	15.56 (4.60)	20.19 (6.87)
	*p*		0.087	0.021	0.001	0.005	0.016	0.002	0.006
Transferrin receptor (mg/L)	Model 1	2139	−0.59 (0.17)	−0.52 (0.17)	−0.62 (0.17)	−0.32 (0.13)	−0.44 (0.12)	−0.30 (0.08)	−0.58 (0.13)
	*p*		0.001	0.004	0.001	0.016	0.001	0.001	<0.001
	Model 2	1688	−0.42 (0.10)	−0.45 (0.10)	−0.52 (0.13)	−0.28 (0.10)	−0.45 (0.11)	−0.30 (0.07)	−0.57 (0.12)
	*p*		<0.001	<0.001	<0.001	0.008	<0.001	<0.001	<0.001

Abbreviations: ln, natural logarithm. Model 1 adjusted for age, gender, ethnicity, smoking, drinking, and BMI. Model 2 adjusted for Model 1 plus oral iron intake, chronic kidney disease, diabetes, and treatment for anemia.

**Table 4:** Linear regression coefficients (SE) of iron status with a unit increase in ln-sum of PFAS in subpopulation, with results weighted for sampling strategy.

	Serum Iron (ug/dL)		UIBC (ug/dL)		TIBC (ug/dL)		Transferrin Saturation (%)		Ferritin (ng/mL)		Transferrin Receptor (mg/L)	
	Adj. β(SE)	*p*	Adj. β(SE)	*p*	Adj. β(SE)	*p*	Adj. β(SE)	*p*	Adj. β(SE)	*p*	Adj. β(SE)	*p*
Gender												
Men	4.07 (2.11)	0.060	−13.71 (4.98)	0.015	−3.53 (3.59)	0.341	3.08 (1.08)	0.012	20.50 (15.29)	0.200	−0.23 (0.09)	0.026
Women	5.37 (1.32)	<0.001	−20.06 (3.66)	<0.001	−13.9 (3.67)	0.002	2.63 (0.71)	0.002	18.81 (7.39)	0.016	−0.74 (0.16)	<0.001
Age, years												
18–40	7.79 (1.75)	<0.001	−20.92 (6.70)	0.007	−10.15 (4.54)	0.041	3.80 (1.21)	0.007	14.94 (8.00)	0.072	−0.51 (0.10)	<0.001
40–59	4.16 (2.42)	0.093	−14.53 (5.23)	0.014	−8.51 (4.01)	0.051	2,12 (1.16)	0.088	42.68 (7.91)	<0.001	−0.87 (0.28)	0.004
≥60	2.32 (1.72)	0.184	−12.81 (3.77)	0.004	−6.19 (3.76)	0.121	2.43 (0.90)	0.016	−7.04 (17.76)	0.697	−0.17 (0.10)	0.114
Ethnicity												
Non-Hispanic white	3.93 (1.67)	0.023	−14.41 (4.59)	0.007	−7.69 (4.11)	0.081	2.24 (0.80)	0.014	18.21 (10.04)	0.081	−0.29 (0.10)	0.009
Other	6.63 (1.33)	<0.001	−22.94 (3.36)	<0.001	−12.10 (1.87)	<0.001	3.96 (0.80)	<0.001	19.08 (12.24)	0.129	−0.93 (0.19)	<0.001
BMI (Kg/m^2^)												
<25	6.78 (2.40)	0.007	−17.98 (6.38)	0.013	−5.42 (5.12)	0.306	4.15 (1.17)	0.003	27.11 (11.25)	0.023	−0.47 (0.10)	<0.001
≥25	4.05 (1.40)	0.006	−16.39 (4.13)	0.001	−10.40 (2.63)	0.001	2.23 (0.94)	0.031	16.62 (8.67)	0.065	−0.58 (0.16)	0.001
Total iron intake (mg/day)												
<13.94	7.07 (1.59)	<0.001	−21.18 (3.87)	<0.001	−7.68 (3.99)	0.073	4.44 (0.46)	<0.001	22.35 (11.27)	0.056	−0.61 (0.09)	<0.001
≥13.94	2.90 (1.73)	0.010	−13.99 (5.85)	0.030	−11.34 (4.72)	0.029	1.23 (1.07)	0.269	23.04 (8.74)	0.013	−0.53 (0.21)	0.014

Adjusted for model 2.

## Data Availability

The datasets analyzed in this study are freely accessible through the NHANES database maintained by the U.S. Centers for Disease Control and Prevention. Data can be retrieved at https://wwwn.cdc.gov/nchs/nhanes/default.aspx (accessed on 8 July 2025).

## Supplementary Materials

The following supporting information can be downloaded at https://www.mdpi.com/article/10.3390/life15081274/s1, Table S1: The Spearman correlation coefficients between different natural logarithm-transformed PFAS concentrations (N = 5050). Table S2: Mean (SD) PFAS levels across three NHANES cycles from 2013 to 2018 in the studied population (N = 5050). Figure S1: Relationship between transferrin receptor and ln-Sum of PFAS fitted by a polynomial regression (quadratic).

## Abbreviations

BMI	Body mass index
Hct	Hematocrit
Ln	Natural logarithm
LOD	Limit of detection
NHANES	National Health and Nutrition Examination Survey
*n*-PFOA	*n*-Perfluorooctanoic acid
*n*-PFOS	n-Perfluorooctane sulfonic acid
RDA	Recommended Dietary Allowance
PFAS	Per- and polyfluoroalkyl substances
PFDeA	Perfluorodecanoic acid
PFHxS	Perfluorohexane sulfonic acid
PFNA	Perfluorononanoic acid
*sm*-PFOS	Perfluoromethylheptane sulfonic acid isomers
TIBC	Total iron-binding capacity
UIBC	Unsaturated iron-binding capacity
