# Supplementary material for: Association Between Serum per- and Polyfluoroalkyl Substances and Iron Status Biomarkers in a Representative Sample of U.S. Adults: NHANES 2013–2018
Source: Life (Basel). 2025 Aug 12;15(8):1274. doi: 10.3390/life15081274 (PMC12387234; doi:10.3390/life15081274)

Supplementary material

Supplemental table S1. The Spearman correlation coefficients between different natural logarithm-transformed PFAS concentrations (N = 5050).

		n-PFOA	*n*-PFOS	*sm*-PFOS	PFNA	PFHxS	PFDeA
n-PFOA	Correlation Coefficient	1.000	0.567	0.673	0.679	0.652	0.451
	P value		<0.001	<0.001	<0.001	<0.001	<0.001
n-PFOS	Correlation Coefficient		1.000	0.821	0.710	0.629	0.639
	P value			<0.001	<0.001	<0.001	<0.001
sm-PFOS	Correlation Coefficient			1.000	0.625	0.756	0.402
	P value				<0.001	<0.001	<0.001
PFNA	Correlation Coefficient				1.000	0.497	0.678
	P value					<0.001	<0.001
PFHxS	Correlation Coefficient					1.000	0.270
	P value						<0.001
PFDeA	Correlation Coefficient						1.000

Supplemental table S2. Mean (SD) PFAS levels across three NHANES cycles from 2013 to 2018 in the studied population (N = 5050)

	2013-2014 N=1676	2015-2016 N=1695	2017-2018 N=1679	
	Mean (SD)	Mean (SD)	Mean (SD)	*P*
PFAS (ng/mL)				
n-PFOA	2.34 (3.17)	1.89 (1.68)	1.70 (1.91)	<0.001
n-PFOS	6.54 (31.84)	5.57 (7.26)	5.11 (6.70)	<0.001
sm-PFOS	2.14 (3.68)	2.12 (1.96)	1.90 (1.83)	<0.001
PFNA	0.90 (0.84)	0.81 (0.74)	0.60 (0.56)	<0.001
PFHxS	2.00 (2.20)	1.71 (1.81)	1.68 (2.47)	<0.001
PFDeA	0.34 (1.37)	0.27 (0.48)	0.29 (0.86)	<0.001

P values were derived from one-way ANOVA comparing PFAS means across the three NHANES cycles.

Supplemental Figure S1: Relationship between transferrin receptor and ln-Sum of PFAS fitted by a polynomial regression (quadratic).

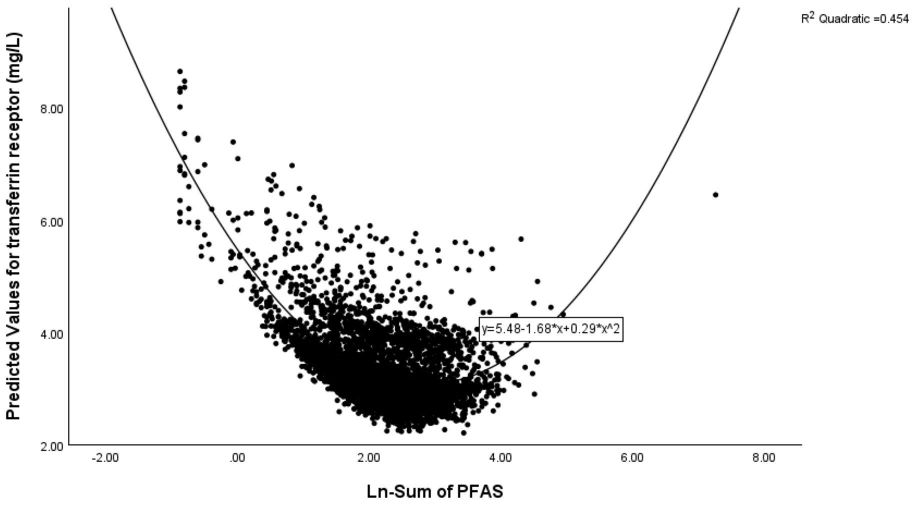

Supplement: Supplementary file 1 [file life-15-01274-s001.zip › life-3790980-supplementary.pdf]
